# Intravascular Ultrasonography (IVUS)—A Tool for Imaging the Eustachian Tube?

**DOI:** 10.3390/bioengineering9120733

**Published:** 2022-11-28

**Authors:** Niels Oppel, Gerrit Paasche, Andre Bleich, Thomas Lenarz, Robert Schuon

**Affiliations:** 1 Department of Otorhinolaryngology, Hannover Medical School, Carl-Neuberg-Str. 1, 30625 Hannover, Germany; 2 Cluster of Excellence Hearing4all, Hannover Medical School, Carl-Neuberg-Str. 1, 30625 Hannover, Germany; 3 Institute for Laboratory Animal Science, Hannover Medical School, Carl-Neuberg-Str. 1, 30625 Hannover, Germany

**Keywords:** Eustachian tube, intravascular ultrasonography, IVUS, imaging, animal model, hyaluronic acid, intraluminal ultrasonography (ILUS)

## Abstract

The Eustachian tube (ET) has a key role in the pathogenesis of otitis media. Until now, there has been a lack of meaningful imaging methods to investigate the ET and its surrounding tissue. The aim of the current study was to investigate the possibilities of imaging the ET using Intravascular Ultrasonography (IVUS). ETs from sheep were scanned ex vivo and in vivo with different IVUS probes. In addition to native ETs, water was also used to improve coupling. Scans were subsequently compared with histological sections and a 3D model of the ET. In addition, ETs with a stenosis induced by a hyaluronic acid depot, after stent insertion, and during lower jaw movement were examined. The IVUS catheter was inserted into the ET lumen without any problems or injuries in all cases. The surrounding structures of the ET were identified in the ultrasound image. In addition, a change in size of the ET lumen due to movement was observed, and the position of the stent and the depot of hyaluronic acid could be examined. With the use of IVUS, a non-invasive possibility to examine the ET over its course with the adjacent structures as well as after different treatments is presented.

## 1. Introduction

The Eustachian tube (ET) is the physiological aerating connection of the middle ear via the nasopharynx and plays an important role as a biomechanical valve. The ET provides pressure equalization, drainage of secretion from the middle ear and protection from retrograde infections, air pressure and sound via the nasopharynx. The epithelial lined ET consists of a smaller bony part, which opens to the middle ear via the protympanum and the functional larger pharyngeal part, which is cartilaginous. The transition from these two parts forms the isthmus, which is a narrowing in the course of the ET [1]. Through the contraction of paratubal muscles, primarily tensor and levator veli palatini muscles, the passively closed ET opens. This happens during actions such as swallowing, yawning and others [2]. Based on these properties, the ET has a key role in the pathogenesis of otitis media [3].

Dysfunction of the ET (ETD) can lead to negative pressure in the middle ear and changed gas composition. These, in turn, lead to a change in the environment of the epithelium or mucoperiosteum, effusion, microbioma or different kinds of chronic middle ear inflammations [4,5]. One of them is otitis media with effusion, which is the most common disease of the ear in children [6]. A patulous ETD results in symptoms such as autophony or feelings of abnormal pressure in the ear when breathing nasally [7]. However, the exact biomechanics of the ET and, thus, also of the ETD are still poorly understood [8]. This statement is found repetitively in various scientific papers and raises the necessity that attention should also be focused on medical imaging, in particular, to develop a better understanding of pathogenesis [5]. Imaging techniques are the basis by which to investigate and better understand the mechanisms of ET. Until now, different techniques have been used to image the ET, such as radiography, computed tomography (CT) [9,10], cone-beam computed tomography (CBCT) [11,12], magnetic resonance tomography (MRI) [13,14], endoscopy [2,15], optical coherence tomography (OCT) [16,17,18] and endoluminal sonography with transfer of Intravascular Ultrasonography (IVUS) used in cardiology [19]. Nevertheless, so far, there is still a lack of clinically suitable examination methods [20]. OCT and IVUS are both characterized in imaging as tomogram. IVUS is characterized by a deeper imaging penetration depth compared to OCT. The OCT has a better resolution, in the range of 10–20 µm, where the IVUS has a resolution of 100–200 µm. The latest attempts and successes in the experimental application were achieved by optical coherence tomography [16,17,18]. Furthermore, the first clinical study was performed with promising results [17]. They also used a transnasal approach, as in the optical coherence tomography study presented earlier [18]. In contrast, Helweg et al. [19] took a peroral approach for their human ex and in vivo IVUS experiments. They concluded that sonography has a high value in vitro as well as in vivo for imaging the ET.

The image characteristics known from B-mode ultrasound diagnostics can also be found in IVUS: tissue-typical echogenicities corresponding to the reflection characteristics, total reflection and extinction, in the case of high impedance differences through air or bone or metal (stent) and artifacts (doubling, diffraction, etc.).

Approximately 20 years later, no further studies using the endoluminal sonography for imaging the ET were to be found. During this time, important developmental steps in the sonography technique were achieved [21].

Decades ago, it was already assumed that inflammation of the middle ear would lead to edema of the mucosa of the ET [22]. While today’s common imaging with CT, CBCT or MRI could easily visualize the usually air-containing middle ear, including the protympanum and soft tissue pathology, this is not adequately visualized in the cartilaginous ET. Here, the IVUS image offers the advantage of an analogous histological section.

Based on this, the aim of the current study was to continue this approach to image the ET via IVUS, but with the further developed technique and by transnasal access. IVUS imaging is being tested ex and in vivo in sheep with differently manipulated ETs.

## 2. Materials and Methods

### 2.1. Ethic Statement

The in vivo experiments in this study were conducted in accordance with the German Animal Welfare Law and the European Directive 2010/63 and approved by the State Office for Consumer Protection and Food Safety, Dept. of Animal Welfare under the number 19/3255. The sheep were housed in the Central Animal Facility (CAF) of Hannover Medical School, and the experiments were performed with regards to the valid directives regarding accommodation, care and usage of experimental animals.

### 2.2. IVUS System

Scans were performed using a Volcano S5 Imaging System (Volcano Corporation, Rancho Cordova, CA, USA) equipped with different catheters and additional devices. The Eagle Eye Platinum catheter (Volcano Corporation) and the Eagle Eye Platinum Short Tip catheter (Volcano Corporation) were used with an electronic phased array. Here, small piezo crystals are placed around the catheter. The system operates at 20 MHz and allows deep penetration and visualization of the tissue. The Eagle Eye Platinum Short Tip catheter has a shorter tip, with a distance of 2.5 mm from the catheter tip to the ultrasound transducer compared to the distance of 10 mm for the Eagle EYE Platinum catheter. Both have a diameter of 3.5 F at the transducer. In some cases, the pullback device R-100 (Volcano Corporation) was used. The device allows the catheter to return over a certain distance with a defined speed. In our experiments, only the speed of 0.5 mm/s was used.

In addition, a Refinity Short Tip catheter (Volcano Corporation) with a diameter of 3 F was used in the experiment. It was connected to the Spinvision PIMR Option Kit (Volcano Corporation), which is characterized by a mechanical rotational principle of a single transducer that rotates at 1800 rpm. With a frequency of 45 MHz, it offers a better resolution but diminished penetration compared to the 20 MHz frequency.

### 2.3. Ex Vivo

This study was performed using seven fresh frozen blackface sheep heads from the abattoir (Hencke Fleischwaren, Bad Bevensen, Germany) or the central animal facility. The heads were defrosted for at least 24 h at room temperature before the scans were performed. For handling reasons, the sheep heads were bisected frozen in the medial sagittal plane with a bone saw (FK 23, Bizerba SE & Co. KG, Balingen, Germany). This facilitated access to the nasopharynx and always ensured perfect visualization of the ET.

To prepare the feasibility test of inserting the IVUS catheter into the ET in vivo, a whole skull was used. A rigid endoscope (HOPKINS^®^ 70°, 4 mm in diameter, 30 cm in length; KARL STORZ SE & CO. KG, Tuttlingen/Germany) attached to a telecam PAL (KARL STORZ) and connected to a Tele Pack Vet X LED RP 100 system (KARL STORZ) was inserted through the meatus nasi ventralis into the nasopharynx, contralateral to the desired ET, to observe the procedure.

To verify the position of the IVUS catheter, CBCT scans (XCat; Xoran^®^, voxel size 0.4 mm, Ann Arbor, MI, USA) were performed before and during the ex vivo experiments.

For the experiment with a stent inside the ET, a stent prototype for the ET of 3–5 mm in diameter and 14 mm in length (bess medizintechnik, Berlin, Germany) loaded onto an application prototype tool (bess medizintechnik) was inserted into the ET orifice up to a defined marker on the instrument. Then, the stent was released, and the instrument removed.

To simulate changes in the tissue surrounding the ET, HA was used as a filler to introduce a stenosing depot in the lateral tube wall. Briefly, following the protocol of Oppel et al., 2022 [23], non-stabilized hyaluronic acid sodium salt from rooster (Sigma-Aldrich Chemie GmbH, Taufkirchen, Germany) was dissolved in isotonic saline solution 0.9% (Isotonic saline solution 0.9% ecoflac plus, B Braun Melsungen AG, Melsungen, Germany) at a concentration of 20 mg/mL and vortexed for 5 min. As a radiographic contrast enhancer, Imeron^®^ 300 (Bracco Imaging Deutschland GmbH, Konstanz, Germany) (0.2 mL/mL) was added direct during the dissolution. This solution was then stored in a 15 mL falcon tube (Cellstar^®^ Tubes, 15 mL, Greiner Bio-One GmbH, Frickenhausen, Germany) for at least 12 h in a fridge and then transferred to 1 mL syringes (SOL-MTM, 1 mL, B Braun Melsungen AG). For some experiments, SonoVue^®^ (Bracco International B.V., Amsterdam, Netherlands) was added to the solution directly before the experiment or was injected pure. A depot with a volume of 0.3 mL up to 1.0 mL was generated in the lateral wall of the ET using a special insertion instrument [23]. During and after this procedure, scans were performed via the IVUS catheter.

To simulate the different movements of the mandible or the soft palate, the corresponding areas were moved manually and repeatedly during the recordings; respectively, the mouth was opened and closed. In another experimental set-up and in the cases of stented ET, the ET was flushed with tap water before a scan was performed. For this, a syringe (Injekt^®^ 5 mL, B Braun Melsungen AG) filled with tap water was used to inject water directly into the ET lumen, until it ran out of the lumen again.

### 2.4. In Vivo

The in vivo experiment was performed with three female mature healthy blackface sheep (provided by the CAF).

The experiments were performed under general anesthesia (GA). The sheep were sedated with midazolam (0.2 mg/kg i.v.; Midazolam-ratiopharm 15 mg/3 mL, ratiopharm GmbH, Ulm, Germany). The GA was then induced with propofol (5–10 mg/mL i.v.; Narcofol^®^ 10 mg/mL, CP-Pharma GmbH, Burgdorf, Germany). Anesthesia was maintained with isoflurane (1.5–2.0% end-tidal inhalation; Isofluran CP 1 mL/mL, CP-Pharma GmbH). Postoperative pain management was provided by carprofen (2 mg/kg i.v.; Carprosol 50 mg/mL, CP-Pharma). The sheep were fixed in a thoracic–ventral position.

The injection of stabilized HA (Restylane^®^ LYFT Lidocaine 1 mL; GALDERMA, Lausanne, Switzerland) was performed following the protocol by Oppel et al., 2022 [23]. Briefly, for local anesthesia, tip swabs soaked with xylometazolinhydrochloride 10 mL (Otriven 0.1%, GlaxoSmithKline Consumer Healthcare GmbH & Co. KG, Munich, Germany) and lidocaine 5 mL (Xylocitin^®^-loc 2%, mibe GmbH, Brehna, Germany) were placed for 5 min in both nostrils. Then, the endoscope was introduced through the meatus ventralis nasalis into the nasopharynx. Using the same method on the other nostril, the injection instrument or the IVUS catheter were also introduced to the nasopharynx.

The IVUS catheter was moved forward into the ET by using a flexible tube with a rigid metal rod and a bendable tip as a guidance catheter. After reaching the ET orifice with the guidance catheter, the metal rod was removed and the Eagle Eye Platinum Short Tip catheter was inserted into the ET through the guidance catheter. The IVUS catheter was then pushed forward manually until a resistance was detectable. By using the pullback device R-100, the IVUS catheter was moved back with a constant speed of 0.5 mm/sec. These scans were performed before and after the injection of HA.

The obtained scans were then compared with the histologic 3D model of the ovine ET by Schuon et al., 2021 [24].

## 3. Results

The IVUS catheter was successfully inserted into the ET in all specimens without effort. Due to a recognizable resistance during insertion of the catheter in the ET, the isthmus was identified. In all experiments, the catheter provoked only a slight opening of the cartilaginous part of the ET. Furthermore, no injury to the structures of the ET was detected in any of the experiments, nor was there any insertion failure into the tubular tissue.

**Figure 1:**
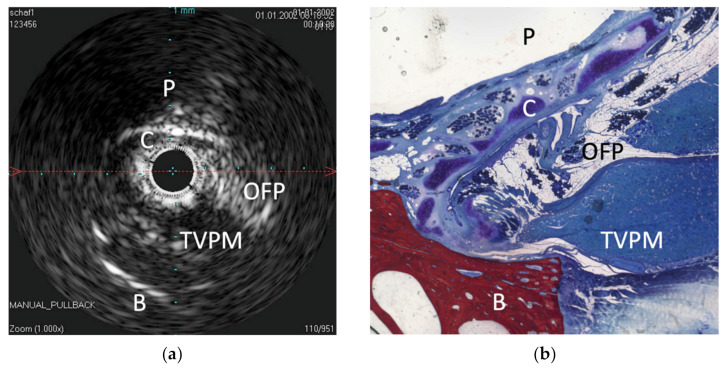
Comparison of the sonographic cross-sectional image near the nasopharyngeal ostium with the histology. (**a**) Sonographic cross-sectional image of the ET lumen near the nasopharyngeal ostium. Note the higher echogenicity of the lining mucosa in the collapsed state of the ET and the sound extinction through bone and air (pharynx). The cartilage (C) is anechogenic. (**b**) Histologic image of this corresponding area. C—cartilage, TVPM—tensor veli palatini muscle, B—bone, P—pharynx, OFP—Ostmann fat pad.

**Figure 2:**
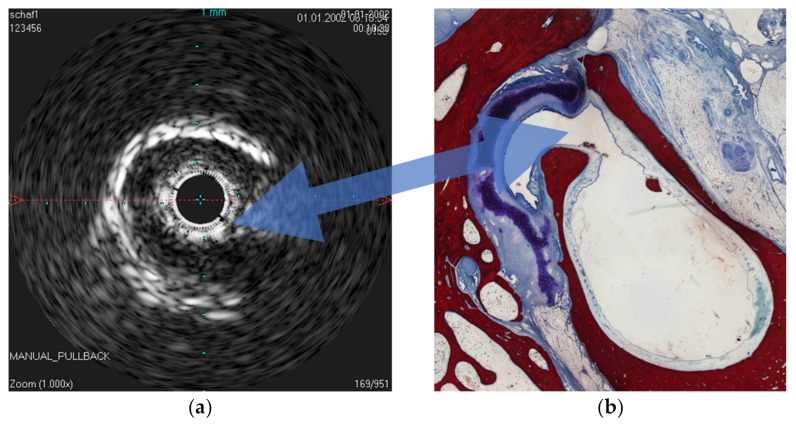
Comparison of the sonographic cross-sectional image in the area of the ET isthmus with the histology. (**a**) Sonographic cross-sectional image of the ET lumen in the area of the isthmus. (**b**) Histologic image of the ET lumen with opening to the recessus hypotympanicus (blue arrow).

**Figure 3:**
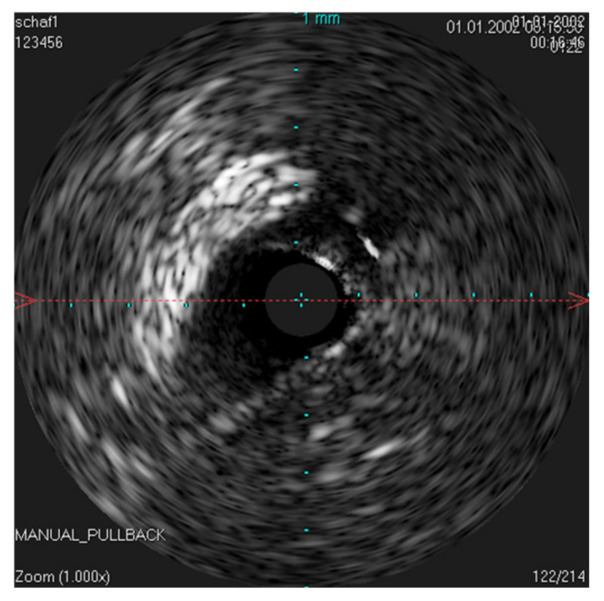
Sonographic image of the ET lumen flushed with water, near to the ET isthmus. The lumen now appears spindle shaped.

The IVUS provided a good image of the slit-formed shape of the ET (Figure 1) and the bony part with the isthmus (Figure 2) in the cross-section view. Bony structures and cartilage can be distinguished from the muscles and the Ostman fat pad. The signal transmission from the catheter to the tissue appeared to be good in all cases, even though the mucosal layer also caused some intense reflections. By flushing the ET with water (Figure 3), the lumen was more visible and became spindle shaped. Reflections at the mucosal layer were reduced.

**Figure 4:**
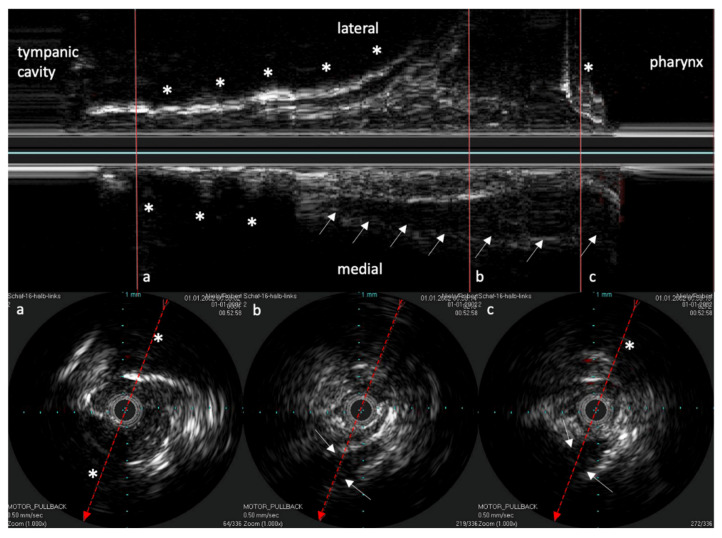
Longitudinal image of the ET with the corresponding cross sections. (**a**) near the ET isthmus. (**b**) in the middle section of the ET. (**c**) near the pharyngeal ostium. Bone is marked with *. The bony structures represent consistent topographic landmarks. The cartilage of the ET shows low echogenicity (white arrows). Note that the dashed red line in the cross-sectional image represents the axis of the longitudinal image. The tip of the arrow points toward the lower part of the longitudinal image.

**Figure 5:**
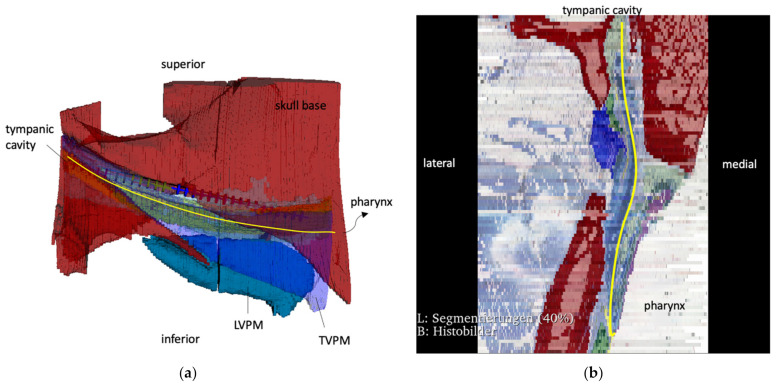
(**a**) Latero-medial 3D view of a reconstruction of an ET from histologic sections. (**b**) Dorso-ventral view of a reconstruction of histological sections; red: bone; turquoise: levator veli palatini muscle (LVPM); blue (slightly transparent): tensor veli palatini muscle (TVPM). The yellow line represents the course of the ET lumen. The bone represents an orienting landmark, as shown in Figure 4.

The reconstructed longitudinal view of the IVUS scans, as shown in Figure 4 with the corresponding circular views, was then compared with the 3D model and the corresponding histological sectional images of Schuon et al., 2021 [24] (Figure 5). Based on this, the surrounding structures could be identified in the IVUS image.

It was possible to analyze the individual cross sections in analogy to histological sections as well as 3D summation of the coherent individual sections, which greatly facilitated the differentiation between image findings and artifacts.

As known from B-scan sonography of the head and neck region, the elastic cartilage of the ET presented echo-poor, and muscular structures were also echo-poor but with more echo-rich structures inside. With a strong impedance jump, bony structures with a total reflection and sound cancellation behind them showed up. The bony structures proved to be helpful for tuning the topography and orientation of the fiber.

The inserted cannula was also well visualized for setting a functional stenosis with HA of the ET, also with high echo signal and sound cancellation.

**Figure 6:**
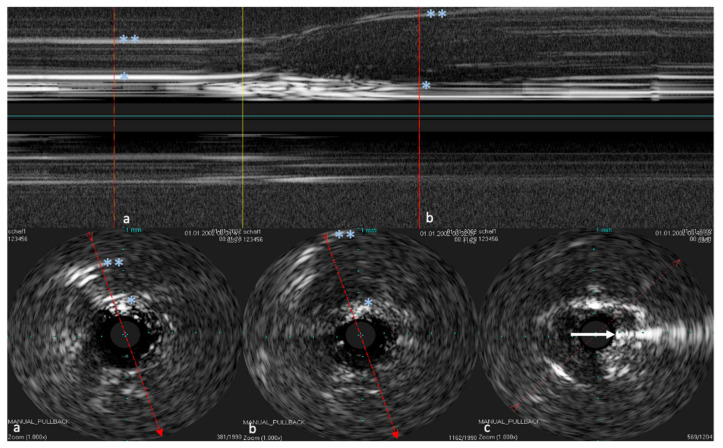
Sonographic image of the injection of HA into the tissue. The probe remained stationary during this procedure. The upper image shows the temporal course of the procedure over approximately 20 s. Image (**a**) is the cross-section of the ET before the injection and image (**b**) during the injection. The yellow line marks the start time of the injection. During the course, how the echogenic tissue layers (marked by the * and **) pressed apart due to the injected echo-poor HA is visible. Image (**c**) shows the cannula (marked by the white arrow) in the sonographic image. Note that image (**c**) is not from the longitudinal view shown above.

Deposition of HA next to the ET was observed using a stationary probe. With a stationary probe, the longitudinal view becomes a timeline. This allows us to observe how the HA is distributed in the tissue and how the depot expands (Figure 6).

By adding the sonography enhancer SonoVue to the HA, a relevant improvement in the visibility of the depot was not achieved.

**Figure 7:**
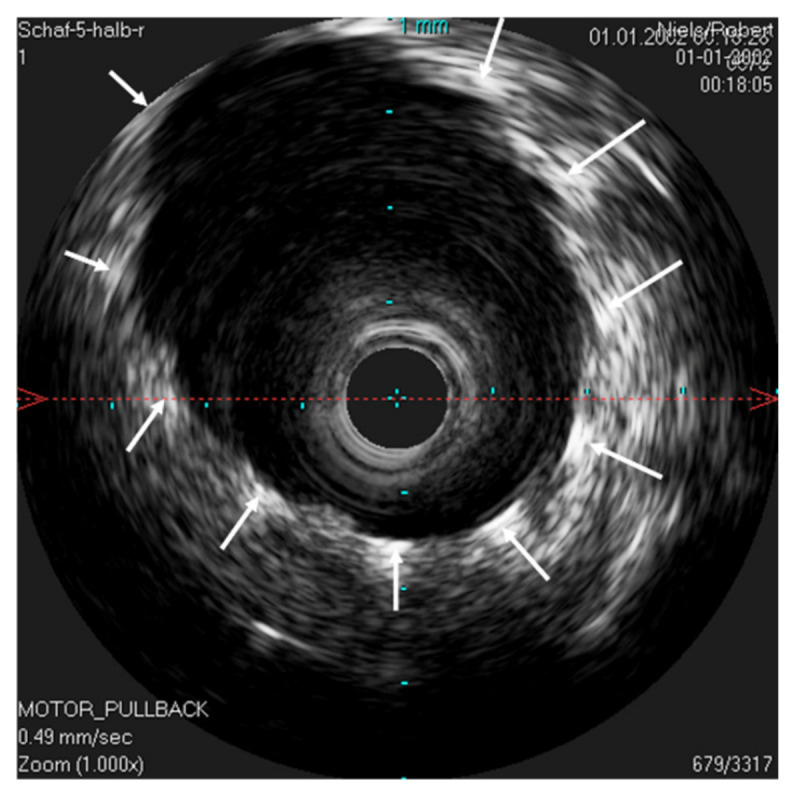
Ultrasound image of the stented ET. The lumen of the ET is opened by the unfolded stent. The IVUS probe is located in the lumen of the stent. The stent struts can be seen echogenically, and sonic extinction is marked with white arrows. Note: the ET was flooded with water before the scan to allow for sonic coupling.

The stent inside of the ET was visualized by the echogenic metal struts of the stent and the widened lumen (Figure 7).

**Figure 8:**
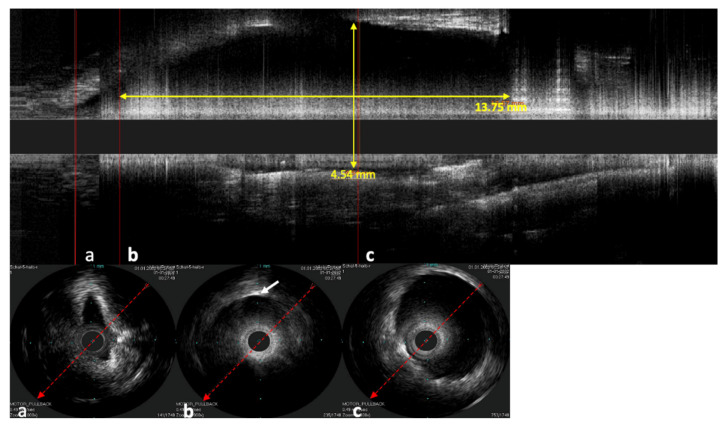
Sonographic image of the stented ET, performed with the Refinity ST probe. The upper image shows the longitudinal view of the scan. Parts (**a**–**c**) show the respective cross-sections of the scans. Part (**a**) shows the area in front of the stent, where the tubal lumen widens toward the stent. Part (**b**) shows the initial area of the stent where the first stent strut can be seen sonographically (indicated by white arrow). Part (**c**) shows the cross-section in the middle section of the stent. The cross-section here was measured to be 4.54 mm. In the longitudinal view, a length of 13.57 mm was measured.

In the longitudinal view, not only can the stent shape be investigated but also how the mucosa of the ET lies around the stent and the position of the stent in the lumen of the ET (Figure 8). In one case, the catheter was next to the stent and not inside of the stent lumen. The stent struts were clearly visible next to the probe in the ultrasound image. Damage to the probe or the stent could not be detected in this case.

**Figure 9:**
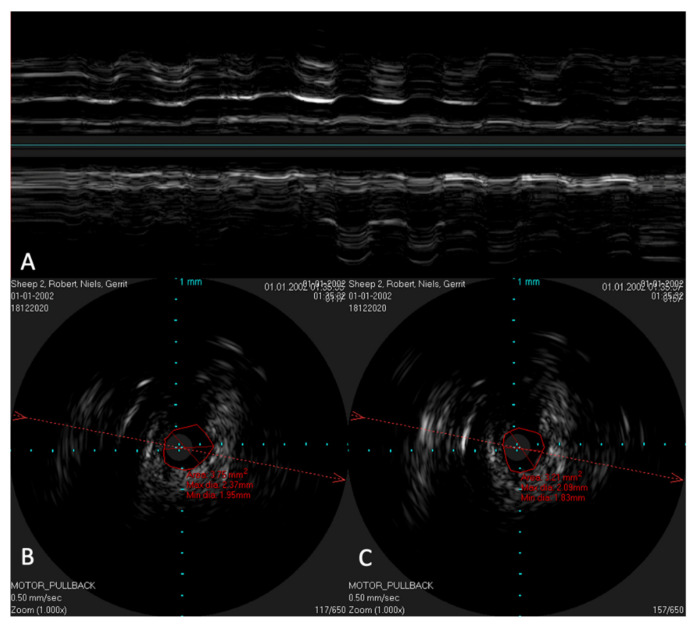
Ultrasound image of the ET with a stationary IVUS probe. During the scan, the lower jaw was repeatedly moved manually, thus opening and closing the sheep’s mouth. Image (**A**) shows the ET over time. The lumen changes and movements of the surrounding structures can be seen as waves. Part (**B**) shows the cross-section of the tube with the mouth open, with an approximate tube lumen of 3.75 mm^2^, where the probe has an area of 1.33 mm^2^. Part (**C**) shows the same area with the jaws closed, where the tube lumen has decreased to 3.21 mm^2^.

In order to possibly investigate the function of the ET, the lower jaw was moved manually during scans with a stationary IVUS catheter in the ET. An extension of the lumen and, thus, a size change of the lumen could be visualized (Figure 9) in the time-based longitudinal view. With the lower jaw closed, a lumen size of 3.21 mm^2^ was measured. The probe itself had a size of 1.33 mm^2^. With the mandible open, this lumen increased to 3.75 mm^2^. In the longitudinal view, this repetitive size change was clearly visible as waves.

**Figure 10:**
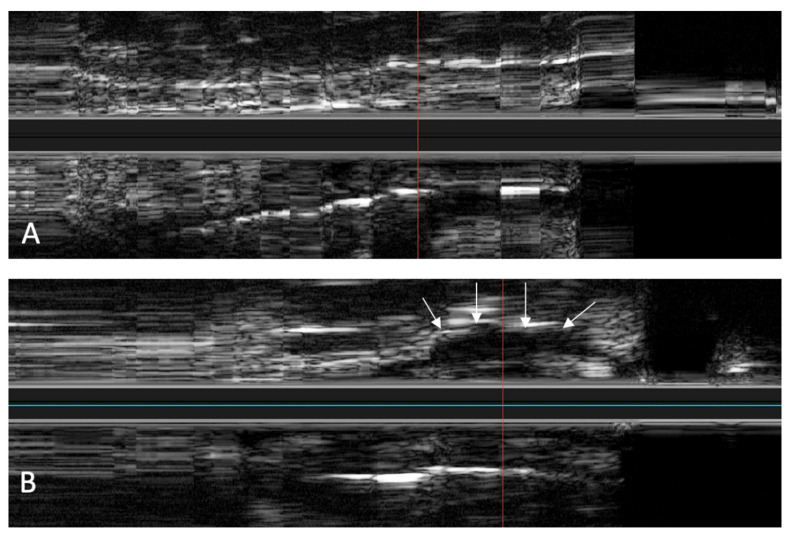
In vivo longitudinal ultrasound image of the ET before (**A**) and after (**B**) placement of an HA depot. In (**B**), the HA depot is anechogenic next to the tubal lumen (marked by the white arrows).

In the in vivo experiments, the IVUS catheter was inserted through the nostrils without any complications. During the insertion of the catheter and the scans, no injuries were detected. The scans were carried out without any problems, both before and after setting an HA depot with 2 mL in the tube course. In a direct comparison of the scans, the depot in the surrounding tissue could also be clearly identified, as shown in Figure 10.

## 4. Discussion

To date, there is still a lack of imaging measures that can adequately represent the causes of ETD in clinical practice. Imaging of the pathological changes in the ET and especially in the mucosa and surrounding structures is the key to a better understanding of the etiology of the disorder and, thus, to the development of therapeutic options. However, in recent years, some promising results have been obtained in imaging the ET using OCT. In this study, in contrast to the predominant current OCT studies [16,17,18], the approach of Hellweg et al. 1996 [19] was revisited to image the ET using an IVUS probe. State-of-the-art probes were used and, in addition, transnasal insertion was performed instead of peroral insertion. Application of an IVUS probe outside the vascular system means that the correct terminology would be intraluminal ultrasonography (ILUS). To be consistent throughout the paper, we have stuck to the term IVUS in all occasions.

IVUS is not the first established method of cardiology that has been transferred for use in the ET. In addition, the nowadays widely used balloon dilatation as a therapeutic option for ETD [25] and the development of a stent as a therapeutic tool to lower the ET opening resistance [26] have to be mentioned.

The method of inserting the catheter into the ET via the nose was proven to be a simple and complication-free method. No complications occurred ex vivo or in vivo in sheep. The short tip of the catheter meant that there was no increased risk to the structures of the middle ear, as previously described in a study with OCT [18]. Compared to oral insertions, no narrow angles have to be overcome for insertion. The catheter can, therefore, be inserted straight into the ET under constant endoscopic control. This not only increases maneuverability and protection against mucosa damage and false insertion but also decreases the forces required to push the probe back and forth. The probe was easy to maneuver in the tube in all attempts and followed the slightly curved course of the ET. In this trial, the insertion depth was indicated by the bony part of the ET. In order to be able to scan an area of the tube that was as large as possible, we prefer the short tip variants of the probes. With these probes, the transducer is only 2.5 mm behind the tip instead of 10 mm, and, therefore, the possibly blind area during the scan is significantly smaller and the risk of damage to the middle ear is reduced. The Eagle Eye Platinum probes work with 64 digital 20 MHz transducers, which offer sufficient spatial resolution and, with a specified maximum of 20 mm image diameter, sufficient depth of image acquisition. In our opinion, a higher frequency would be able to display more details and would be desirable in the future. In comparison, the Refinity ST works with a 45 MHz transducer. This is mechanically rotated by the Spinvison PIMR in the catheter. Due to the higher frequency, this probe achieves an even more precise spatial resolution, which is accompanied by a lower penetration depth of 14 mm. We could achieve good images with both probes, although the mechanical rotation in the Refinity ST probe caused it to vibrate slightly, making the live image more unstable.

The scan of the native ET achieved good results, although minor artifacts occurred, probably because the sound coupling was disturbed by air bubbles. By flushing the ET with water, it was possible to produce a consistently good image, indicating a better coupling. At the same time, the tube unfolded and the lumen appeared spindle-shaped. Scanning the ET in vivo without adding a coupling substance resulted in scans of good quality. This could probably still be improved if a sterile liquid, for example, physiologic saline or ultrasound gel, could be instilled into the ET by means of a catheter. Care should be taken that no pathogens are introduced and that the middle ear is not flooded. Otherwise, there is a risk of infections of the middle ear and ruptures of the tympanic membrane.

The placement of a stenosis of the ET by means of a hyaluronic acid depot in the lateral paratubal space could be followed live with the ultrasound by an increased area of low echogenicity. The position of the cannula in the tissue could be determined at the beginning, and then the injection process could be followed. Thus, it was possible to see how echogenic tissue layers moved further away from each other and the echo-poor HA spread in between. The HA depot was subsequently seen as a contiguous circumscribed echo-poor area between echo-rich tissue layers. In addition, the position of the depot could be determined by comparison of scans from before and after the injection of HA. Mixing the HA with SonoVue did not improve the display of the depot in our experiments and, thus, did not result in any measurable gain. Probably, the mixing ratio was not chosen appropriately, but this would need further trials to confirm. In summary, it can be stated that by means of the IVUS probe, not only can the injection process be controlled more precisely, but also the position of the depot can be determined immediately afterwards. In particular, ultrasound-guided navigation of the cannula could provide considerable safety during interventions in paratubular soft tissue. Formerly fatal consequences of injections due to puncturing the internal carotid artery [27] could be reduced by additional control using IVUS.

The stent placed in the ET could be clearly imaged using ultrasound after flushing the ET with water. Instead of water, ultrasonic gel or similar could also be used to ensure sound coupling. Thus, immediately after stenting the ET, the position of the stent can be verified without the use of radiography. This is a direct advantage for the patient and the treating physician. Based on this, it is expected that IVUS can also be used in follow up investigations when tissue might have formed on top of the stent. Due to the slit-shaped ET structure, the situation is different to the circular vessel in cardiology. Therefore, placement in the slit-shaped area outside the stent is more probable and occurred in one of our attempts. This suggests that stent control with IVUS might be more difficult than in cardiology. No dislocation of the stent was observed, after all.

IVUS is a promising imaging modality for investigating the ET. As OCT, it requires the insertion of a probe into the tubal lumen. While the ultrasound probe emits sound, the OCT probe emits light. The advantages and disadvantages of both methods result from the different physical properties of light and sound. The near-infrared light, with the short waves, offers a very high spatial resolution with a lower penetration depth of 1–3 mm. In comparison, ultrasound at a frequency of 20–45 MHz, as commonly used in IVUS, offers a lower spatial resolution but a significantly deeper penetration depth of 5–10 mm [28]. To pair the strengths of both techniques, the combination of IVUS and OCT [29,30] could be an interesting way to visualize ET [29,30].

Altogether, this study has shown that IVUS can be used to visualize the ET with its surrounding structures. This allows for better control of possible treatments for the ET. However, a better resolution would be desirable in order to be able to assess changes, such as those in the mucosa, more precisely.

## 5. Conclusions

In the ET, the application of IVUS was performed with transnasal insertion of the probe and without penetration of the lining mucosa. IVUS, as an endoluminal imaging technique, offers the possibility of demonstrating the integrity of the mucosa of an ET over its entire course. Structures with an impedance jump, such as bony structures or metal stents with fine struts, are easy to detect due to total reflection and acoustic shadowing. Tissue structures such as cartilage, musculature or fat could also be assigned due to their typical tissue-specific echogenicity. With the probe in a static position, both movements of the ET by manipulation of the lower jaw and the ultrasound-guided placement of an injection cannula for setting a depot next to the ET were depicted under direct image view, which could be important for the future application of fillers in a patulous tube.

## Data Availability

All the data that support the findings of this study are available on request from the corresponding author.
